# Spatial Analysis of Factors Influencing Long-Term Stress in the Grizzly Bear (*Ursus arctos*) Population of Alberta, Canada

**DOI:** 10.1371/journal.pone.0083768

**Published:** 2013-12-27

**Authors:** Mathieu L. Bourbonnais, Trisalyn A. Nelson, Marc R. L. Cattet, Chris T. Darimont, Gordon B. Stenhouse

**Affiliations:** 1 Spatial Pattern Analysis and Research Lab, Department of Geography, University of Victoria, Victoria, British Columbia, Canada; 2 Canadian Cooperative Wildlife Health Centre, Western College of Veterinary Medicine, University of Saskatchewan, Saskatoon, Saskatchewan, Canada; 3 Applied Conservation Science Lab, Department of Geography, University of Victoria, Victoria, British Columbia, Canada; 4 Foothills Research Institute, Hinton, Alberta, Canada; University of Regina, Canada

## Abstract

Non-invasive measures for assessing long-term stress in free ranging mammals are an increasingly important approach for understanding physiological responses to landscape conditions. Using a spatially and temporally expansive dataset of hair cortisol concentrations (HCC) generated from a threatened grizzly bear (*Ursus arctos*) population in Alberta, Canada, we quantified how variables representing habitat conditions and anthropogenic disturbance impact long-term stress in grizzly bears. We characterized spatial variability in male and female HCC point data using kernel density estimation and quantified variable influence on spatial patterns of male and female HCC stress surfaces using random forests. Separate models were developed for regions inside and outside of parks and protected areas to account for substantial differences in anthropogenic activity and disturbance within the study area. Variance explained in the random forest models ranged from 55.34% to 74.96% for males and 58.15% to 68.46% for females. Predicted HCC levels were higher for females compared to males. Generally, high spatially continuous female HCC levels were associated with parks and protected areas while low-to-moderate levels were associated with increased anthropogenic disturbance. In contrast, male HCC levels were low in parks and protected areas and low-to-moderate in areas with increased anthropogenic disturbance. Spatial variability in gender-specific HCC levels reveal that the type and intensity of external stressors are not uniform across the landscape and that male and female grizzly bears may be exposed to, or perceive, potential stressors differently. We suggest observed spatial patterns of long-term stress may be the result of the availability and distribution of foods related to disturbance features, potential sexual segregation in available habitat selection, and may not be influenced by sources of mortality which represent acute traumas. In this wildlife system and others, conservation and management efforts can benefit by understanding spatial- and gender-based stress responses to landscape conditions.

## Introduction

Spatial patterns of species decline and extinction have been linked to complex interactions among anthropogenic factors, such as habitat loss and fragmentation [1]–[3], over-exploitation [4], climate change [5], [6], and competition with invasive species [7], [8]. Whereas many large predators lack the behavioural plasticity necessary to adapt to rapid change [9], at greatest risk are large bodied predators with diminished geographic range, small population size, low fecundity, and which occupy higher trophic levels [4], [10], [11]. To date, efforts to examine spatial patterns of species decline have focused primarily on changing patterns of species distributions, abundance, and mortality in response to anthropogenic activities and habitat fragmentation [12]–[14]. Although such studies provide essential understanding about how wild populations have responded to changing environments, they are generally retrospective due to a temporal disconnect between the disturbance event and associated population decline [15]. What effective policy intervention requires are real-time measures of potential stressors with associated spatial methods to reliably understand where individuals within populations might be at most risk of declines.

Recently, measures of the physiological response of wildlife to external stressors are emerging as a viable approach for analyzing contemporary impacts of habitat conditions and disturbance on the health and fitness of individuals and populations [15], [16]. Cortisol concentrations measured from saliva, blood, feces, and hair [17]–[19] have been used as a non-invasive approach to quantify long-term stress responses in animals such as the northern spotted owl [20], squirrel gliders [21], ungulates [22]–[24], wolves [25], and grizzly bears [26], [27]. Vertebrates respond to noxious external stimuli by activation of the hypothalamic-pituitary-adrenal axis with the resultant release of glucocorticoids, such as cortisol, into the blood circulation [28] allowing the organism to respond to the stressor with the goal of maintaining or re-establishing homeostasis [29]. However, persistent or repeated exposure to stressful stimuli, and resultant continued circulation of glucocorticoids, have been found to impair immune system performance, increase susceptibility to disease, and decrease growth and reproductive capacity in some species [15], [28], [30].

An emblematic species of western North America, the grizzly bear (*Ursus arctos*) has experienced substantial reduction in its historic range due to human settlement and development [31]. Conservation of remaining localized populations is difficult due to conflicting public opinion and land-use [32]. Grizzly bears in Alberta, Canada, have recently been estimated to number fewer than 700 individuals [33], and as a result the provincial population was listed as *Threatened* in 2010 [34]. Grizzly bears in Alberta occupy a landscape heavily impacted by human activities and resource extraction. Industrial activities (e.g., forestry, oil and gas exploration, mining, and agriculture) and extensive road networks are prevalent throughout grizzly bear habitat within the province resulting in a highly fragmented multi-use landscape [35], [36]. Although a number of parks and protected areas exist, many are subject to a wide variety of recreational pursuits and high human visitation rates. Anthropogenic land-use and open road access features represent primary causes of grizzly bear mortality as bears have been found to select anthropogenic disturbed habitats to exploit seasonal food availability, which has increased contact with humans [35], [37]–[39]. Further, patterns of land-use have resulted in genetically fragmented sub-populations that may not be viable in the long-term [36], [40]. Less clear is how these activities, which vary spatially and in their character, affect the physiology of individuals.

Spatial patterns of anthropogenic land-use, forest conditions, and topography also influence the distribution of available resources (e.g., [38]). Conceptually, habitat quality accounts for the range of conditions that have an impact on the health of an animal occupying the habitat [41]. Grizzly bears in Alberta occupy large home ranges (ranging from approximately 300 km^2^ for females to upwards of 1500 km^2^ for males) allowing them to seasonally select a diverse assemblage of habitat types necessary to meet their nutritional needs [42], [43]. True to their description as opportunistic omnivores, the diet of grizzly bears in Alberta is generally low in protein consisting mostly of green vegetation, fruits, and insects [38], [44], although the consumption of ungulates varies seasonally [45]. Accounting for spatial variability in environmental factors related to habitat, such as forest conditions, landcover, vegetation productivity, and elevation, that influence the availability of food resources and have an impact on the health of individuals is an important consideration when assessing the relationship between landscape conditions and physiology [46], [47].

We know that human impacts to the landscape, including habitat loss and alteration [33], contribute to grizzly bear mortality [48]–[50]. Yet, little is understood regarding how human activities and habitat conditions affect bears physiologically. Given small grizzly bear population sizes [36], densities, and reproductive rates [51], research on the interaction between physiological status and landscape conditions is essential. Although physiological status can be represented by a wide range of metrics, we specifically focus on long-term stress in this study because of growing recognition that long-term stress is an important factor linking ecological change with impaired health and population performance in wildlife [52], [53]. Accordingly, our goal is to quantify spatial relationships between landscape conditions and long-term stress levels in grizzly bears by statistically integrating a spatially and temporally broad dataset of hair cortisol concentrations (HCC) with data representative of habitat conditions and anthropogenic disturbance. To meet this goal we address the following objectives:

1. Quantify impacts of habitat conditions and anthropogenic landscape disturbance on observed spatial patterns of HCC levels in Alberta grizzly bears.
2. Develop a spatially explicit model to predict HCC levels across grizzly bear habitat based on current landscape conditions.
3. Interpret the spatial distribution of predicted HCC values using data describing the relative importance and security of grizzly bear habitat.

To address these objectives, novel spatial methods are required to integrate HCC data with spatially continuous data representing environmental conditions and to quantify observed relationships. Methods presented here, including marked point pattern analysis using kernel density estimation and non-parametric regression using random forests, provide an effective means for analyzing ecological data and are appropriate for future research on wildlife and HCC.

### Study Area

**Figure 1:**
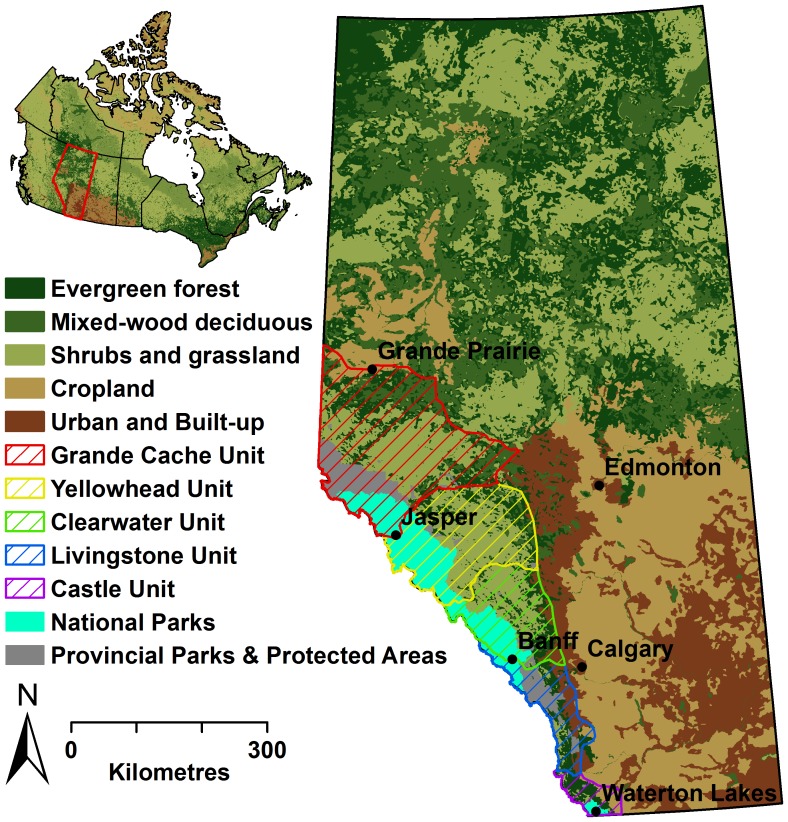
Study area location in Alberta, Canada. Grizzly bear hair samples were collected in each bear management unit during a single summer (Yellowhead – 2004; Clearwater – 2005; Livingstone – 2006; Castle – 2007; Grande Cache – 2008).

The study was carried out for five grizzly bear management units (BMUs) in Alberta, Canada (Figure 1). Representing an area of nearly 111,000 km^2^, the Grande Cache, Yellowhead, Clearwater, Livingstone, and Castle BMUs are divided by major east-west transportation corridors. As such, the BMUs largely represent genetically isolated populations, although some inter-population movement does occur [36]. Due to the geographic extent of the study area, vegetation, topography, and local weather conditions are highly variable. Elevation ranges from 450 m to 3500 m and increases from east to west. In the western mountainous region, habitat types include alpine and sub-alpine ecosystems comprised of fir (*Abies* spp.), pine (*Pinus* spp.), and spruce (*Picea* spp.), and wet-meadow complexes [54], [55]. To the east, lower elevation foothills comprised of mixed-wood forests of pine, aspen and poplar (*Populus* spp.), spruce, and balsam fir (*Abies balsamea*) represent a transitional zone between the Rocky Mountains and the prairies [54]. Mean temperatures range from 12°C in the summer to −7.5°C in the winter, and mean annual precipitation is 450–800 mm. Major grizzly bear foods found in the region include herbaceous plant growth such as sweetvetch (*Hedysarum* spp), Canada buffaloberry (*Shepherdia canadensis*), bearberry (*Arctostaphylos uva-ursi*), horsetail (*Equisetum* spp), dandelions (*Taraxacum officinale*), and clover (*Trifolium* spp), as well as a variety of insects and ungulates [38], [56].

Resource extraction activities within the BMUs also vary spatially. They include forestry, oil and gas exploration, mining, and agriculture. To service resource extraction activities, an extensive network of roads exists that provide access to grizzly bear habitat resulting in increased human-bear conflict and mortality [49], [50], [57]. These roads and areas are also widely used for recreation, including hunting, fishing, hiking, and trail-riding with all-terrain vehicles and snowmobiles. A network of parks and protected areas, including Jasper, Banff, and Waterton National Parks, as well as a number of provincial parks and wilderness reserves, which generally exclude resource extraction activities, are also found throughout the BMUs.

## Materials and Methods

### Ethics Statement

Grizzly bear hair collection was undertaken as part of an initiative to conduct a population inventory program for the species. The population inventory work was carried out at the request of the Government of Alberta (Environment and Sustainable Resource Development) by the Foothills Research Institute’s Grizzly Bear Program (FRIGBP) and followed the techniques described by Woods et al. [58] and Proctor et al. [59]. Grizzly bears are not provincially endangered and were not a protected species during the time when this sampling occurred. All samples were collected on provincial and federal lands under the authority of the Government of Alberta (Environment and Sustainable Resource Development and Alberta Parks) and the Government of Canada (Parks Canada). This inventory program was approved by the Alberta Department of Sustainable Resource Development animal care committee and by Parks Canada when sampling occurred in their jurisdiction in each year of data collection. Annual research permits and animal care approvals were obtained from both provincial and federal agencies responsible for permits and licensing of these activities.

### Hair Cortisol Concentrations

Cortisol concentrations (picograms per milligram of hair - pg/mg) were measured in grizzly bear hair samples obtained annually from 2004 to 2008. As cortisol accumulates in hair for the duration of its growth, the HCC values used in this analysis represent a stress signal from the period of hair growth during the year preceding hair sample capture. Hair is a stable medium that can be collected non-invasively from free-ranging animals [26]. It can be transported and stored with relative ease (e.g., paper envelope at room temperature) and substances incorporated into growing hair, including cortisol, remain detectable for years to centuries [60]. These attributes make hair cortisol concentrations particularly effective for quantifying long-term stress in far-ranging species such as grizzly bears [26], [61]. Hair samples were collected using barbwire hair snags [55], [56] randomly placed within 7 km × 7 km grid cells and repositioned at 14 day intervals throughout known grizzly bear habitat in each BMU during the spring and early summer (see [62]–[66] for methods used to collect grizzly bear hair samples in each BMU). A total of 304 HCC values (*n*  = 168 females, *n*  = 136 males) were extracted from hair samples collected in the five BMUs (see [26] for details regarding procedures to extract cortisol concentrations from grizzly bear hair). As only two of the 304 HCC values were from the same individual, the HCC data represent an effective characterization of long-term stress in the Alberta grizzly bear population. The range of observed HCC values (0.16–14.94 pg/mg vs. 0.16–23.66 pg/mg) and mean HCC ±95% CI (1.20±0.29 pg/mg vs. 1.52±0.34 pg/mg) were similar between males and females, respectively. Further, comparison between the mean HCC values for males and females did not indicate a statistically significant difference (independent samples *t*-test with observed values: *p*  = 0.19; independent samples *t*-test with ln-transformed values: *p*  = 0.13).

### Habitat Conditions

We represented grizzly bear habitat conditions by integrating a variety of spatial data. These variables, characterizing forest conditions, landcover, topography, and vegetation productivity measured using remotely sensed data, represent proxies for food availability (see Table 1 for rationale for inclusion of variables). Percent crown closure and percent conifer were modelled and scaled from 0% to 100% through classification of Landsat Thematic Mapper (TM) 5 and Landsat 7 Enhanced Thematic Mapper Plus (ETM+) imagery informed by topographic derivatives from a digital elevation model (DEM) [67]. Landcover was classified into eight classes: upland trees, wetland trees, upland herbs, wetland herbs, shrubs, water, barren land, and snow/ice, using Landsat TM 5 and ETM+7 imagery, and topographic derivatives [68], [69]. We assessed terrain conditions using a DEM detailing elevation for the study area obtained from the Government of Canada spatial data portal Geobase and resampled to 1 km grid cells. A terrain ruggedness index, providing a measure of terrain complexity and variability [70], and a compound topographic index, which represents potential soil moisture based on slope, catchment area, and upstream water sources [71], were derived from the DEM.

**Table 1:** Variables used to predict HCC levels in grizzly bears.

Abbreviation	Variable	Range	Rationale
**Habitat conditions**			
cc	Percent crown closure (%)	0–100	Influences forest understory vegetation abundance
pctcon	Percent conifer (%)	0–100	Correlated with herbaceous food abundance
lcover	Landcover (categorical)	1–8	Proxy for presence and abundance of food sources
dhi_cum	Dynamic Habitat Index – cumulative greenness (unitless)	0.33–18.50	Estimate of total vegetation productivity
dhi_cv	Dynamic Habitat Index – coefficient of variation (unitless)	0.19–1.35	Estimate of seasonal change in vegetation productivity
dhi_min	Dynamic Habitat Index – minimum cover (unitless)	0–0.40	Lowest estimated annual vegetation productivity
elev	Elevation (m)	450–3500	Impacts landcover, vegetation cover, and human access
tri	Terrain ruggedness index (unitless)	0–189.33	Impacts human access and grizzly bear mortality
cti	Compound topographic index (unitless)	3.86–18.03	Correlated with herbaceous foods and presence of ungulates
**Habitat selection**			
rsf_s1	Resource Selection Function – hypophagia (categorical)	0–10	Probability of habitat selection following den emergence
rsf_s2	Resource Selection Function – early hyperphagia (categorical)	0–10	Probability of habitat selection during the summer
rsf_s3	Resource Selection Function – late hyperphagia (categorical)	0–10	Probability of habitat selection during the fall
rsf_max	Resource Selection Function – maximum value (categorical)	0–10	Maximum observed habitat selection across all three seasons
**Anthropogenic disturbance**			
rd_dd	Roads – distance decay (unitless)	0–1	Impacts human access and contribute to landscape fragmentation
rail_dd	Railways – distance decay (unitless)	0–1	Contribute to grizzly bear mortality
wl_dd	Oil and gas well-sites – distance decay (unitless)	0–1	Concentrated sites of human activity and contribute to habitat fragmentation
ln_den	Secondary linear features – density (km/km^2^)	0–7.28	Contribute to habitat fragmentation, density of forest edges, and impacts human access
cblk_l	Forest harvest blocks – ≤ than 15 years old (% cut/km^2^)	0–100	Younger seral forests have greater abundance of herbaceous foods
cblk_g	Forest harvest blocks –>than 15 years old (% cut/km^2^)	0–100	Food availability decreases as time since disturbance increases
pa	Proportion parks and protected area (unitless)	0–1	Less disturbance compared to surrounding landscape

We used indices from the Dynamic Habitat Index (DHI) [72], [73], which has been linked to observed spatial patterns of avian species [74], biodiversity gradients [75], and home range size of carnivores [76], to characterize vegetation productivity in the study area. The DHI indices are calculated from remotely sensed imagery and summarize annual trends in monthly images of the fraction of photosynthetically active radiation (fPAR). In this study, fPAR is derived from Advanced Very High Resolution Radiometer (AVHRR) reflectance values with a spatial resolution of 1 km from 2003 to 2007 [72], [77]. The DHI is comprised of three indices representing vegetation productivity: cumulative greenness, variation in greenness, and minimum cover [72], [78]. Cumulative greenness, which represents total vegetation productivity, is estimated annually by summing monthly fPAR observations. Variation in greenness, representative of seasonal changes in productivity, and consequently the seasonal availability of food resources, is calculated using the coefficient of variation in fPAR values over a year. Highly seasonal landscapes, such as alpine environments, where greenness values vary substantially due to snowpack, receive higher values than regions that are productive year round, such as evergreen forests [72]. The minimum cover is an estimate of the lowest level of vegetative productivity available year round and may influence the persistent use of habitat by herbivorous species. As production of leafy biomass and fruits consumed by grizzly bears is seasonally dependent [45], [79], regions with high seasonality and cumulative greenness, as well as high minimum cover, are likely representative of high quality grizzly bear habitat.

### Habitat Selection

We characterized spatial patterns of seasonal habitat use by grizzly bears throughout the study area using resource selection functions (RSFs) where the probability of habitat use is ranked from 0 (low) to 10 (high) [43], [80]. An RSF models the probability of use of a resource, relative to its availability, based on occurrence patterns of an animal on the landscape [81]. The RSFs developed for this study area are based on third order selection [82], and as such the modelled probability of habitat use by grizzly bears is representative of selection at the patch level (see [43] for RSF model details and accuracy). Separate RSF models were used for male and female bears in three seasons, hypophagia (1 May to 15 June), early hyperphagia (16 June to 31 July), and late hyperphagia (1 August to 15 October), as well as the maximum observed RSF value for all three seasons (Table 1).

### Anthropogenic Influence

Anthropogenic features we considered included roads, railways, oil and gas well-sites, cut-lines, power-lines, pipelines, and forest harvest blocks (Table 1). Anthropogenic data were provided by Alberta Sustainable Resource Development and are mapped and updated by the FRIGBP based on appearance of disturbance features in Landsat imagery. We represented roads, railways, and oil and gas well-sites using an exponential distance decay function, *e^−ad^* where *d* is the distance in metres to the feature and *a* was fixed at 0.002 [80]. The distance decay surfaces decrease linearly from a value of 1 at the location of the anthropogenic feature to a value of 0 at a distance of approximately 2000 m. We represented secondary anthropogenic linear features, such as cut-lines, power-lines, and pipelines, as a cumulative linear density per 1 km^2^ grid cell (km/km^2^).

We divided forest harvest blocks into two age classes (≤15 years old; >15 years old) to account for regeneration and resultant differences in grizzly bear food availability present within the harvested areas [38], [83]. We assessed the influence of forest harvest blocks by calculating the proportion of harvested area in 1 km^2^ grid cells. Resultant grid cells ranged from a value of 1 representing an area that has been completely harvested to 0 indicating no harvesting had occurred in the area. Finally, we modelled the influence of the parks and protected areas in the region, which represent a noted contrast in terms of land-use compared to the surrounding landscape, based on proportion parks and protected area within a 10 km radius from 1 km^2^ grid cells. Values ranged from 1 for cells completely within parks and protected areas to 0 when no parks and protected areas were in the immediate vicinity.

### Conservation Areas and Habitat Security

Core and secondary grizzly bear conservation areas, based on observed patterns of grizzly bear occurrence, resource availability, and road density, have been mapped within the five BMUs [80]. Core conservation areas, which are meant to act as a population source, are defined as regions with high quality habitat (using RSF scores as surrogates) and road densities below 0.6 km/km^2^. Secondary conservation areas also contain high quality habitat, however road densities are higher at 1.2 km/km^2^ which increases the risk of mortality and decreases the population source capacity of the area. Within these regions, habitat states have been assessed by characterizing the landscape based on habitat quality and mortality risk [84]. In this analysis we incorporate three defined habitat states in 1 km^2^ grid cells: secure habitat (low mortality risk), sink habitat (high mortality risk), and non-critical habitat. Combined with the present network of parks and protected areas, the core and secondary conservation units as well as the observed habitat states were used to help interpret geographic relationships in the modelled distribution of HCC values within the five BMUs.

### Kernel Density Estimation of Hair Cortisol Concentrations

To integrate the HCC point data with spatially continuous habitat and anthropogenic variables, we converted HCC data from points to a continuous surface using kernel density estimation (KDE). KDE is frequently used in wildlife analyses to summarize spatial variation in the intensity of habitat selection over large areas (e.g., the utilization distribution) based on telemetry point data [85]. KDE is also employed to spatially allocate attribute data (based on a statistically or ecologically significant bandwidth) over an area representative of the sample locations to understand how underlying environmental characteristics influence spatial pattern [86]. The KDE surface provides greater detail regarding the spatial variability of the underlying point pattern, which is the case in the spatial distribution of HCC levels. Kernel density estimation of a marked point pattern is defined by
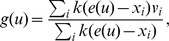
where *k* is a Gaussian kernel, the HCC point data values are given by 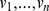 at locations 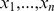, *u* are the smoothed HCC values, and 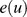 is an edge correction factor based on the reciprocal of the kernel mass



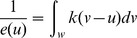
inside the observation window or spatial extent *W* [87], [88]. The spatial extent was defined separately for males and females in each BMU as the minimum convex polygon (MCP) of HCC sample locations [89]. Kernel values were stored in a 1 km cell matching the spatial resolution of landscape variables. A 9 km kernel bandwidth was defined as it corresponds to the average daily area used by an adult female grizzly bear [43] and was supported by least-squares cross-validation, which identifies the bandwidth that minimizes the summed squared error between observed values and smoothed values.

We assessed the influence of individual HCC points on the stability of the male and female KDE surfaces by generating 99 leave-one-out bootstrap HCC kernel density surfaces for validation [90]. We quantified uncertainty in the HCC surfaces for each BMU by determining the proportion of KDE pixels that fell within a 95% confidence interval (*p<*0.01) of similar observed values in the bootstrap surfaces [91]. We compared the distribution and central tendency of the values in the KDE HCC surfaces to the original HCC values using Kolmogorov-Smirnov and Mann-Whitney U tests.

### Random Forest Models

We used random forest models to quantify the influence of habitat and anthropogenic variables on the spatial distribution of male and female grizzly bear HCC values from the KDE surfaces. A non-parametric recursive regression method, random forests combine multiple regression trees built using bootstrap samples of data [92]. Each individual regression tree is grown to its maximum size using random subsets of predictor variables [93]. Trees are combined by averaging and estimation of response values is performed using the withheld out-of-bag observations [93], [94]. The model variance explained is assessed based on the accuracy of the prediction of out-of-bag data. Random forests have been found to be ideally suited to ecological data as they do not require linear relationships, effectively model variable interactions, can handle missing data and correlated variables, are more stable than traditional regression trees to minor changes in input data, and have high predictive power [92]–[94]. Variable importance in random forest models is quantified using two complementary output metrics. The first is a normalized comparison of the mean square error of model predictions with predictions generated using randomly permuted predictor values from the out-of-bag data [93]. The second is the average total decrease in node impurity attributed to splitting on each variable measured using the residual sum of squares and provides an indication of node prediction accuracy attributed to each variable.

We ran random forest models for both male and female grizzly bears using a random subset of 50% of the available data. The remaining 50% of the data were withheld for model validation. Each model included 1000 trees to allow stabilization of out-of-bag error and 18 variables were randomly selected for consideration at each split. Due to varying landscape conditions and anthropogenic influence inside and outside of parks and protected areas, we produced secondary random forest models with an inside parks and protected areas/outside parks and protected areas distinction to explore potentially differing variable importance.

We used validated random forest models to predict male and female HCC values in 1 km^2^ grid cells for the area of the five BMUs outside the confines of the MCPs used in the development of the KDE surfaces. We explored relationships between the 10 most influential variables and the predicted HCC values by summarizing the mean predictor values that corresponded with the lower (0.16–0.45 pg/mg: low HCC), inner (0.46–1.62 pg/mg: moderate HCC), and upper (>1.62 pg/mg: high HCC) quartiles of the input HCC point data. We used the pooled interquartiles of the input male and female HCC values in order to facilitate direct comparison of HCC levels between the sexes. These same HCC value breaks were used to summarize the percentage of pixels within the area of each BMU classified as a low, moderate or high HCC.

To aid interpretation of predicted HCC values, we assessed the associations between HCC values and parks and protected areas, core conservation areas, and secondary conservation areas based on the frequency distribution of male and female HCC values occurring within each of these management units. We also assessed relationships between the predicted male and female HCC values and classified secure, sink, and non-critical habitat types using frequency distributions based on 1 km^2^ pixel associations.

## Results

### Validation of HCC Kernel Density Layers

Comparison of input HCC values and generated KDE HCC surfaces indicated that the KDE represented the range and spatial distribution of HCC values. Greater than 80% of pixels in the HCC KDE surfaces for all five BMUs fell within the 95% confidence interval (*p*<0.01) when compared to values in the 99 leave-one-out bootstrap surfaces. Kolmogorov-Smirnov and Mann-Whitney U tests showed the attributes of HCC KDE surfaces were not significantly different than measured HCC values in each of the BMUs (Table 2).

**Table 2:** HCC kernel density estimation validation results by bear management unit (BMU).

BMU	Proportion data within 95% CI (*p*<0.01)	Kolmogorov-Smirnov	Mann-Whitney U
Castle	0.89	*p* = 0.07	*p* = 0.09
Livingstone	0.86	*p* = 0.12	*p* = 0.17
Clearwater	0.88	*p* = 0.09	*p* = 0.08
Yellowhead	0.88	*p* = 0.16	*p* = 0.14
Grande Cache	0.87	*p* = 0.21	*p* = 0.27

### Male HCC Models

**Figure 2:**
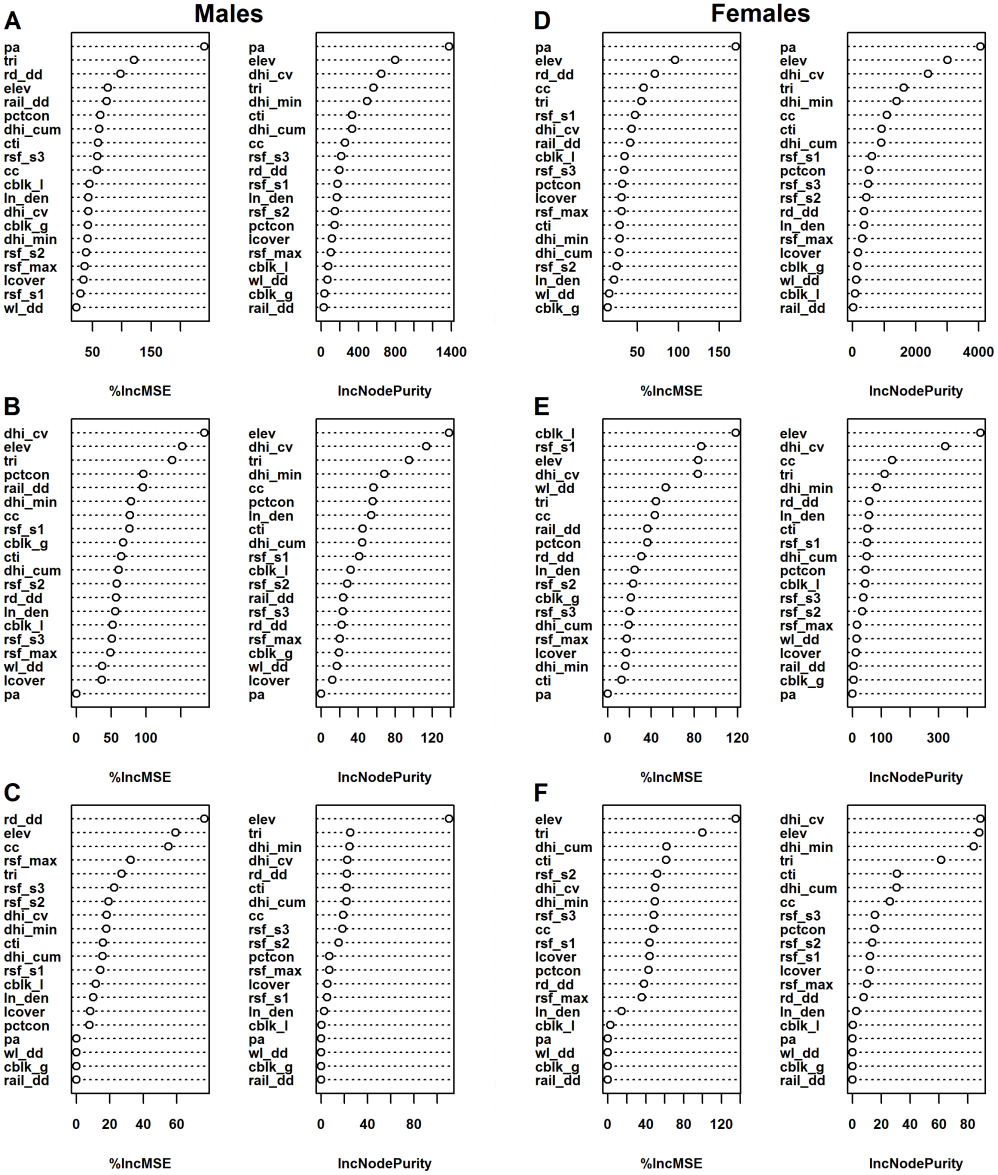
Variable importance metrics for male and female HCC random forest models. Variable importance for the male (A) total model, (B) outside parks and protected areas model, and (C) inside parks and protected areas model, as well as the female (D) total model, (E) outside parks and protected areas model, and (F) inside parks and protected areas model. Variable importance plots on the left of each panel (%IncMSE) represent the accuracy of random forest model predictions based on regression tree splits made using each variable. Plots on the right of each panel (IncNodePurity) indicate how often each variable was used as a split in regression trees aggregated through the random forest. For example, in panels A & D the proportion parks and protected areas was selected often as a tree split in the random forest and had a high predictive HCC value accuracy.

Using random forest metrics, habitat conditions and anthropogenic disturbance variables considered explained 74.28% of the variance in the male HCC data (MSE  = 0.17). Validation of the male model using the withheld data returned an *r^2^* of 0.71. The male total model predicted a mean HCC value of 0.89 pg/mg (±95% CI: 0.01 pg/mg; range: 0.17 pg/mg –3.25 pg/mg). Proportion parks and protected areas was the most influential variable in the male model (Figure 2A). Habitat condition variables with the greatest influence included the three topographic metrics (elevation, terrain ruggedness index, and compound topographic index), the DHI metrics (cumulative greenness, variation in greenness, and minimum cover), as well as crown closure and the late hyperphagia RSF. Influential anthropogenic variables included distance decay to roads, and to a lesser extent distance decay to railways and the density of secondary linear features (Figure 2A). Mean variable values associated with low, moderate, and high predicted HCC values revealed generalized trends in the relationship between variables and HCC values (Table 3). Male HCC values had an inverse relationship with the proportion parks and protected areas. HCC levels were low-to-moderate at high and low elevations, and low stress-levels were associated with rugged terrain and low soil wetness. Observed HCC levels increased as the distance decay to roads decreased and as forest crown closure increased. DHI metrics showed male stress levels were lowest in areas with greater seasonality, low minimum cover and moderate cumulative greenness. The late hyperphagia RSF had the strongest relationship with male HCC values among the habitat selection variables considered. Spatial associations showed male HCC levels increased with increasing incidence of habitat use in the fall season.

**Table 3:** Mean values of the 10 most influential variables in the total random models associated with lower, mid, and upper quartiles of the predicted HCC levels in male and female grizzly bears.

Variable	HCC range (pg/mg)		
	0.16–0.45	0.46–1.62	>1.62
**Males**			
pa	0.85	0.13	0.17
elev	2164.92	1277.83	1690.15
rd_dd	0.01	0.56	0.36
tri	33.87	10.67	21.32
dhi_cv	0.71	0.43	0.41
dhi_min	0.04	0.11	0.14
dhi_cum	6.02	11.11	10.13
cti	6.62	14.12	10.80
cc	17.22	43.66	48.76
rsf_s3	3	4	6
**Females**			
pa	0.01	0.12	0.68
elev	940.91	1427.64	1980.69
rd_dd	0.69	0.55	0.12
tri	3.88	12.15	29.49
dhi_cv	0.43	0.53	0.65
dhi_min	0.11	0.11	0.06
dhi_cum	11.47	8.42	6.54
cblk_l	0.03	0.02	0.01
cc	47.10	39.54	30.76
rsf_s1	2	5	6

Evaluation of the influence of differing landscape conditions inside and outside parks and protected areas on the modelled HCC values altered the importance of predictor variables. The male grizzly bear outside parks and protected areas model explained 55.34% of the variance in the HCC data (MSE  = 014; *r^2^* = 0.72). The mean predicted male HCC for the outside parks and protected areas model was 1.01 pg/mg (±95% CI: 0.01 pg/mg; range: 0.21 pg/mg –3.25 pg/mg). Excluding the influence of parks and protected areas increased the importance of density of secondary linear features and proportion of forest harvest blocks greater than 15 years old (Figure 2B). The male inside parks and protected areas model explained 74.96% of the variance (MSE  = 0.07, *r^2^* = 0.84), similar to the total model. The inside parks and protected areas model mean predicted male HCC was 0.55 pg/mg (±95% CI: 0.02 pg/mg; range: 0.17 pg/mg –2.88 pg/mg). While the influence of variables related to habitat quality, such as elevation, terrain ruggedness, crown closure, and DHI metrics, were similar to the total model, the importance of distance decay to roads increased substantially for males when the spatial extent of the model was restricted to parks and protected areas (Figure 2C).

**Figure 3:**
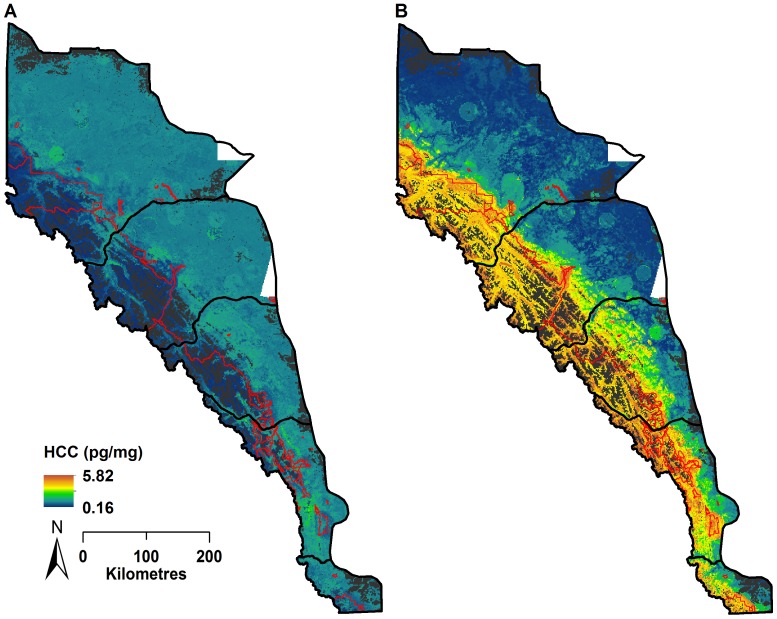
Geographic distribution of the predicted HCC levels from gender-specific total random forest models. Predicted HCC values for (A) male and (B) female grizzly bears. Parks and protected areas are shown in red. Regions of non-habitat (e.g., rock and ice) are shown in grey.

**Figure 4:**
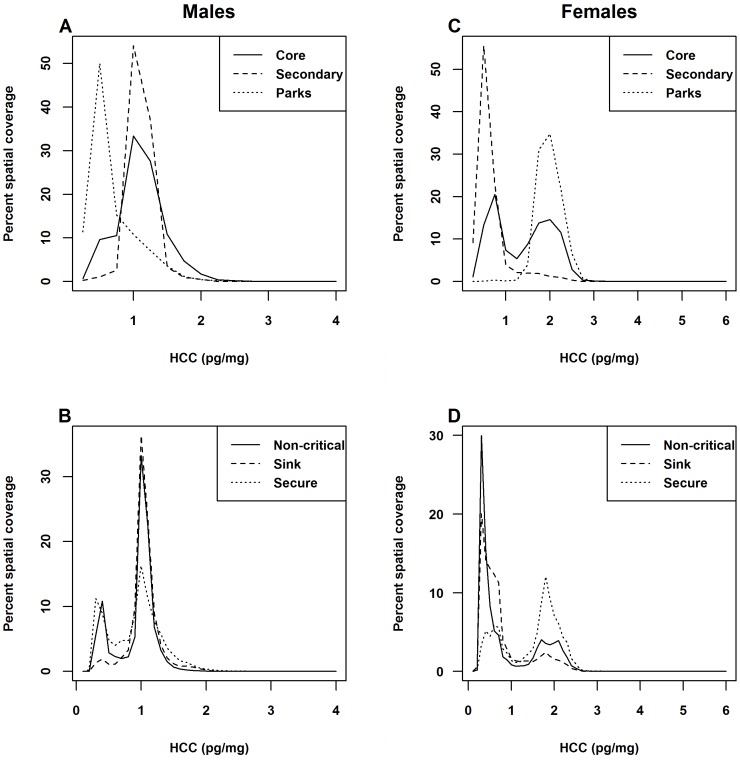
Frequency distributions of predicted HCC values associated with conservation management units and habitat states. Percent spatial coverage of predicted HCC values associated with (A – males; C – females) parks and protected areas, core conservation areas, and secondary conservation areas, as well as (B – males, D – females) secure, sink, and non-critical habitat states.

HCC values predicted using the total male random forest model showed spatial patterns of long-term stress in males were generally low in western national parks and increased in non-protected and low elevation regions to the east and south (Figure 3A). The highest HCC values for males were restricted to zones in and around smaller protected areas, while low and moderate HCC values were more frequent and continuously distributed on the landscape (Table 4). Frequency distributions of predicted HCC values associated with parks and protected areas, core conservation areas, and secondary conservation areas revealed similar trends. Low male HCC values had more frequent spatial associations with parks and protected areas (Figure 4A). Regions designated as core conservation areas had a higher frequency of moderate male HCC values, while secondary conservation areas were associated with moderate-to-high male HCC values (Figure 4A). In terms of secure, sink, and non-critical habitats, moderate-to-high male values were more frequently associated with non-critical and sink habitats (Figure 4B).

**Table 4:** Percent area of the bear management units (BMU) and study area classified as low, moderate, and high HCC based on the geographic distribution of predicted male and female HCC values.

BMU	Low HCC(0.16–0.45 pg/mg)		Moderate HCC(0.46–1.62 pg/mg)		High HCC(>1.62 pg/mg)	
	Male (%)	Female (%)	Male (%)	Female (%)	Male (%)	Female (%)
Castle	2.93	6.83	95.67	58.90	0.73	33.58
Clearwater	22.17	10.22	75.92	49.34	1.76	40.61
Grande Cache	13.04	51.86	83.47	26.96	0.91	18.60
Livingstone	11.54	0.54	78.53	42.61	8.37	55.29
Yellowhead	13.04	34.37	74.60	27.95	1.30	34.77
Total study area	16.11	33.89	79.90	33.53	1.88	30.47

### Female HCC Models

Total female random forest models explained 68.46% (MSE  = 0.21; *r^2^* = 0.70) of the variance in female HCC values. The mean predicted female HCC from the total model was 1.01 pg/mg (±95% CI: 0.02 pg/mg; range: 0.16 pg/mg –5.82 pg/mg). Variable importance was similar to the total male model, as proportion parks and protected areas was again the most influential variable (Figure 2D). Important habitat variables in the total female model included the three topographic metrics, the three DHI metrics, crown closure, the hypophagia RSF, and percent conifer. Important anthropogenic variables included distance decay to roads and railways, as well as the proportion of forest harvest blocks less than 15 years old. Generally, relationships between the mean values of the 10 most influential variables associated with low, moderate, and high predicted female HCC values contrasted those in the male model (Table 3). Female HCC values increased as the proportion parks and protected areas, elevation, and terrain ruggedness increased. Unlike males, low female HCC levels were associated with areas closer to roads, areas which have a higher density of recent forest harvest blocks, and forests with moderate crown closure. DHI metrics showed females had lower HCC levels in areas exhibiting less seasonality and higher minimum cover and cumulative greenness. Hypophagia RSF values had the strongest associations with female HCC levels which increased as the probability of habitat use increased in the spring season.

The female outside parks and protected areas model had a higher variance explained (67.61%; MSE  = 0.20; *r^2^* = 0.71), compared to the female inside parks and protected areas model (58.15%; MSE  = 0.15; *r^2^* = 0.58). The mean female HCC was 0.73 pg/mg (±95% CI: 0.02 pg/mg; range: 0.16 pg/mg –5.82 pg/mg) for the outside parks and protected areas model and 1.86 pg/mg (±95% CI: 0.02 pg/mg; range: 0.40 pg/mg –3.10 pg/mg) for the inside parks and protected areas model. Compared to the male model, the change in variable importance was more pronounced in the female outside parks and protected areas model as the influence of proportion of forest harvest blocks less than 15 years old, distance decay to railways, and distance decay to oil and gas well-sites all increased (Figure 2E). However, unlike the male inside parks and protected areas model, restricting the female model to landscape conditions within parks and protected areas decreased the influence of all anthropogenic variables while increasing the influence of the DHI and topographic metrics (Figure 2F).

Spatial patterns of the predicted long-term stress levels for female grizzly bears contrasted predicted male HCC patterns. Predicted female HCC values were generally low in the eastern portions of the study area where anthropogenic disturbance is concentrated and increased substantially in the foothills and high elevation parks and protected areas in the west (Figure 3B). As a result, low female HCC values were more frequently associated with secondary conservation areas and sink habitats (Figure 4C; Figure 4D). High, moderate, and low predicted female HCC values were more evenly distributed on the landscape, and high female values had a far greater geographic representation compared to similar male HCC values (Table 4). Similar to males, the Livingstone BMU had the greatest percent area with high female HCC values, while the Grande Cache BMU had the greatest concentration of low male and female HCC values (Table 4).

## Discussion

Grizzly bears in Alberta, Canada face many challenges as a result of increasing anthropogenic activities and habitat fragmentation throughout their current range. While knowledge regarding causes of mortality, habitat use, and spatial patterns of habitat fragmentation and loss is considerable, less is known regarding the impacts of landscape conditions on the health of grizzly bears [26]. We found no obvious differences in observed input HCC values between males and females. Similarity in male and female input HCC levels suggests that baseline cortisol levels are robust to differences in the types and timings of life history events. However, the spatial distribution of predicted HCC values, based on random forest models integrating HCC stress surfaces with environmental covariates, differed between males and females. In general, female HCC levels appeared to follow a gradient based on elevation with moderate-to-high values predicted in the mountain parks and core conservation areas, and low-to-moderate values predicted in lower elevation forests, prairies, and aspen parkland. The pattern for male HCC values also appeared to follow a similar gradient. Male HCC values were predicted to be low in the mountain parks and moderate in the prairies and aspen parkland located in the eastern portion of the study area. In contrast to females, focal areas of high HCC values were not evident for males.

Gender based variation in spatial patterns of HCC levels suggest the type and intensity of stressors are not uniform across the landscape. Results suggesting females, and to a lesser extent males, have low-to-moderate long-term stress levels in response to environmental conditions present in regions highly impacted by humans appear counterintuitive. However, disturbance features related to resource extraction activities (e.g., forest harvest blocks, roads, pipelines), which represent the dominant forest disturbances in the study area due to extensive fire suppression [95], [96], have been correlated with the presence and abundance of foods consumed by grizzly bears [38], [97], [98]. Findings presented here support the hypothesis that long-term stress levels in certain wildlife species may be correlated with the availability and abundance of foods. Examining glucocorticoid levels detected in grizzly bear scat in the Yellowhead BMU, Wasser et al. [99] found preliminary evidence that grizzly bear stress levels were generally low in areas with high densities of anthropogenic disturbance and higher in areas with fewer disturbances. They hypothesized that observed patterns of stress may be related to the greater availability and distribution of foods in anthropogenic disturbance features. Similar inverse relationships between glucocorticoid levels and food availability and diet have been observed in other grizzly bear populations [27], [100], as well as in a number of bird [47] and marine species [101]. For grizzly bears in Alberta, with a diet comprised largely of seasonally dependent herbaceous food sources [45], optimizing nutritional uptake over a short summer season is essential to ensure energy demands are met over the winter. Consequently, areas with more abundant food sources may present a low-stress environment for grizzly bears despite human activities.

While male and female stress levels in highly industrialized areas in the east were comparable, the high mountain parks in the west represented notable broadly dispersed areas of high stress for females and low stress for males. The observed differences in HCC levels may be the result of gender-specific differences in exposure to stressors and/or differences in perception and response to stressors both inside and outside of parks and protected areas. For example, Graham et al. [57] found females were more likely to select habitat associated with roads and to cross roads during the day, while males avoided roads and were more likely to cross them at night. Similarly, females have been found to select edge habitats associated with anthropogenic disturbance while male habitat selection is more frequently associated with naturally occurring edges [97]. As a result, outside of parks and protected areas the higher density of anthropogenic disturbance features, which are generally avoided by males, may explain the slightly higher observed male HCC levels compared to values inside the parks and protected areas where anthropogenic activities are more localized. However, as males occupy very large home ranges (upwards of 1500 km^2^) they may be more capable of mitigating the cumulative effects of external environmental stressors compared to females whose home ranges are smaller (approximately 300 km^2^) and have less inter-annual variability. This may explain the lower spatial variability in HCC levels observed in males across the study area.

Compared to males, female exposure to external stressors elicits strong stress levels inside parks and protected areas. Despite the importance of parks and protected areas as wildlife refuges, Gibeau et al. [102] classified nearly half of the available habitat in these areas as unsuitable for grizzly bears (i.e., high elevation areas with poor food availability and concentrated areas of human recreation and activity). As a result, the availability of high quality habitat and foods is restricted in these regions and may result in higher observed stress levels in females. We propose the same conditions do not elicit a strong stress response in males due to sexual segregation of high quality habitat selection within parks and protected areas. Females have been found to make greater use of sub-optimal habitats and areas in close proximity to humans to avoid males and reduce the risk of infanticide when they are with cubs [36], [57], [103]–[107]. Female habitat selection is also philopatric as female offspring tend to occupy home ranges that take in part of, or are in close proximity, to the home ranges of their mother [59], [106], [108]–[110]. If males are dominating or excluding female use of quality habitat in parks and protected areas the resulting disparity in resources available to each gender could help explain observed geographic differences in the stress levels in parks and protected areas.

Further, in an assessment of grizzly bear body condition across the study area, Cattet et al. [111] found bears inside mountainous parks had poorer body condition compared to bears outside parks and protected areas, and suggested observed patterns may be linked to relatively low food availability in high mountain parks. If food availability is linked to long-term stress in grizzly bears the differences in body condition may in fact be the result of observed differences in stress levels inside and outside of parks and protected areas and warrants further study. While the observed spatial associations between high stress levels and increased incidence of habitat selection in the hypophagia and late-hyperphagia RSF layers provide preliminary evidence of potential impacts of competition on long-term stress, future studies explicitly considering impacts of sexual segregation and conspecific competition on stress in grizzly bears would also be of interest.

The observed spatial patterns of long-term stress in areas with high densities of human disturbance and activity represent a potential grizzly bear management opportunity. Numerous studies have proposed restricting human access may help reduce human-induced grizzly bear mortality (e.g., [49], [50]). We suggest managing human access to grizzly bear habitat, for example by closing roads following the conclusion of resource extraction activities or restricting access to high quality habitat in parks and protected areas, could lead to health benefits for individuals, particularly females. As external environmental stressors can negatively impact reproduction [30], the ability of females to occupy low-stress environments with abundant food sources may help ensure the long-term viability of populations. However, potential physiological gains afforded by food resources associated with anthropogenic disturbed habitats may be offset if grizzly bears continue to experience high rates of human-induced mortality in these areas (humans are responsible for upwards of 80% of observed grizzly bear mortality in the area [48], [49], [112]).

While one might expect habitats associated with high rates of mortality to elicit a strong long-term stress response, human-caused grizzly bear mortalities represent events characterized by acute traumas and not the result of prolonged continued exposure to stressors. Consequently, we hypothesize that observed low-to-moderate stress levels associated with high-risk (e.g., high chance of mortality) high-reward (e.g., food availability) habitat suggests a willingness to risk human contact to optimize foraging opportunities. Moreover, the observed stress profiles might also signal a degree of habituation to such contact that potentially puts grizzly bears at greater risk of mortality in low-stress environments. Future research should consider whether observed long-term stress levels influence the probability of grizzly bear mortality in human influenced landscapes.

## Conclusion

We have shown that the geographic dissimilarity in long-term stress levels of grizzly bears appears to be both context-dependent [21], and similar to northern spotted owls [20], gender specific. Similar to black bears [46] and grizzly bears in other regions [27], [100] the availability and ability to procure resources appears to impact spatial patterns of the modelled stress levels in Alberta grizzly bears. Our results also reflect findings in species such as squirrel gliders [21], ungulates [22], [24], and wolves [25], where the measured stress levels varied according to the density, distribution, and perhaps most importantly, the nature of anthropogenic disturbance and activities throughout their habitat. The methodology we present here extends an aspatial glucocorticoid metric to a spatially local representation of potential long-term stress in grizzly bears based on current environmental conditions. The information provided highlights spatial variability in the long-term stress levels of male and female grizzly bears. Future directions in this and other wildlife systems include modelling the impacts of individual behaviour and the effects of interaction on stress, predicting changes in long-term stress based on future disturbance patterns and climate change, and exploring spatial relationships between stress and body condition. Our methods may also be applicable in spatial analyses of point sampled stress metrics taken from other far ranging wild animals (e.g., polar bears [113]). Finally, while our models are specific to the grizzly bear population in Alberta, many populations in western North America occupy landscapes with similar environmental stressors. Consequently, our findings may offer an indication of similar interactions in other regions. Future species conservation efforts should therefore attempt to better understand gender and spatial based differences in the physiological response of wildlife to landscape conditions.
